# Supplementary material for: A mechanoelectrical mechanism for detection of sound envelopes in the hearing organ
Source: Nat Commun. 2018 Oct 9;9:4175. doi: 10.1038/s41467-018-06725-w (PMC6177430; doi:10.1038/s41467-018-06725-w)
Supplement: Supplementary file 1 — Supplementary Information [file 41467_2018_6725_MOESM1_ESM.pdf]

Supplementary Information

A mechanoelectrical mechanism for detection of sound envelopes in the hearing organ

Nuttall et al

Supplementary Note

The effects of the envelope on the current through the MET channels is described in Fig. 7 in the main text. However, the spectra of the currents revealed an additional peak at 2f1-f2 (oblique arrow in Suppl. Fig. 1a). The amplitude of this peak changed as the envelope moved from a "peaky" shape to a flatter one (Suppl. Fig. 1b shows how the amplitude at 2f1-f2 depended on the center-tone phase). Note that the current tends to be smaller for center-tone phase 90°. Measurements parameters were the same as in Fig. 7.

These data prompted a study of the 2f1-f2 spectral peak in the model described in the main text (Fig. 7e – g). For 1-nm stimuli, the amplitude of the 2f1-f2 peak was around 40 dB below the amplitude of f1 (Suppl. Fig. 2a). For larger stimuli, the amplitude at 2f1-f2 increased but the dip near center-tone phase 90° remained. Hence, the model reproduced aspects of the finding shown in Suppl. Fig. 1b.

In the model, we also studied how the amplitude of different distortion products varied with stimulus level and bundle resting position (X_0). For non-saturating stimuli, distortion at f_e and $2f_e$ was maximal for values of X_0 ranging from 5 to 10 nm (Suppl. Fig. 2b and c). At this bundle offset, distortion at the frequency 2f1-f2 was minimal. For stimuli large enough to saturate the MET current, the behaviour was however qualitatively different (Suppl. Fig. 2d). For such stimuli, the 2f1-f2 peak showed no minimum and the f_e and $2f_e$ peaks grew with increasing X_0.

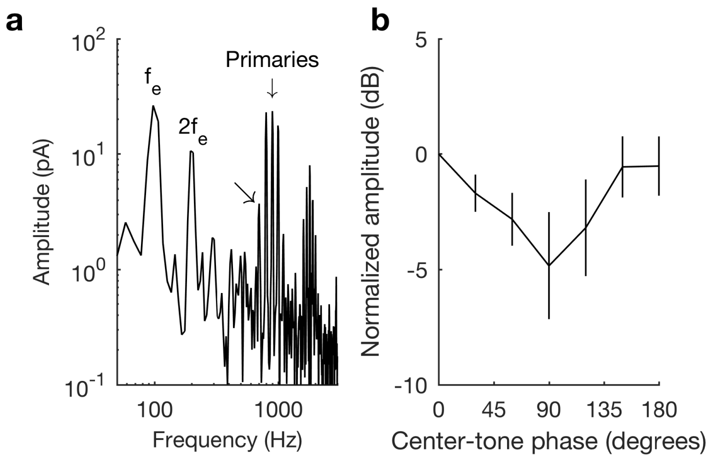

Suppl. Fig. 1. Distortion in patch-clamp recordings. a. Example spectrum of the MET current in response to three-tone stimulation with center-tone phase 0°. Responses at f_e and $2f_e$ are described in the main text. Oblique arrow denotes the 2f1-f2 peak, the amplitude of which is shown in panel b. Holding potential, -84 mV. **b**. Normalized amplitude of the spectral peak at 2f1-f2 ± standard error of the mean across 9 cells. The average current for phase 0° was 4.9 ± 1.7 pA.

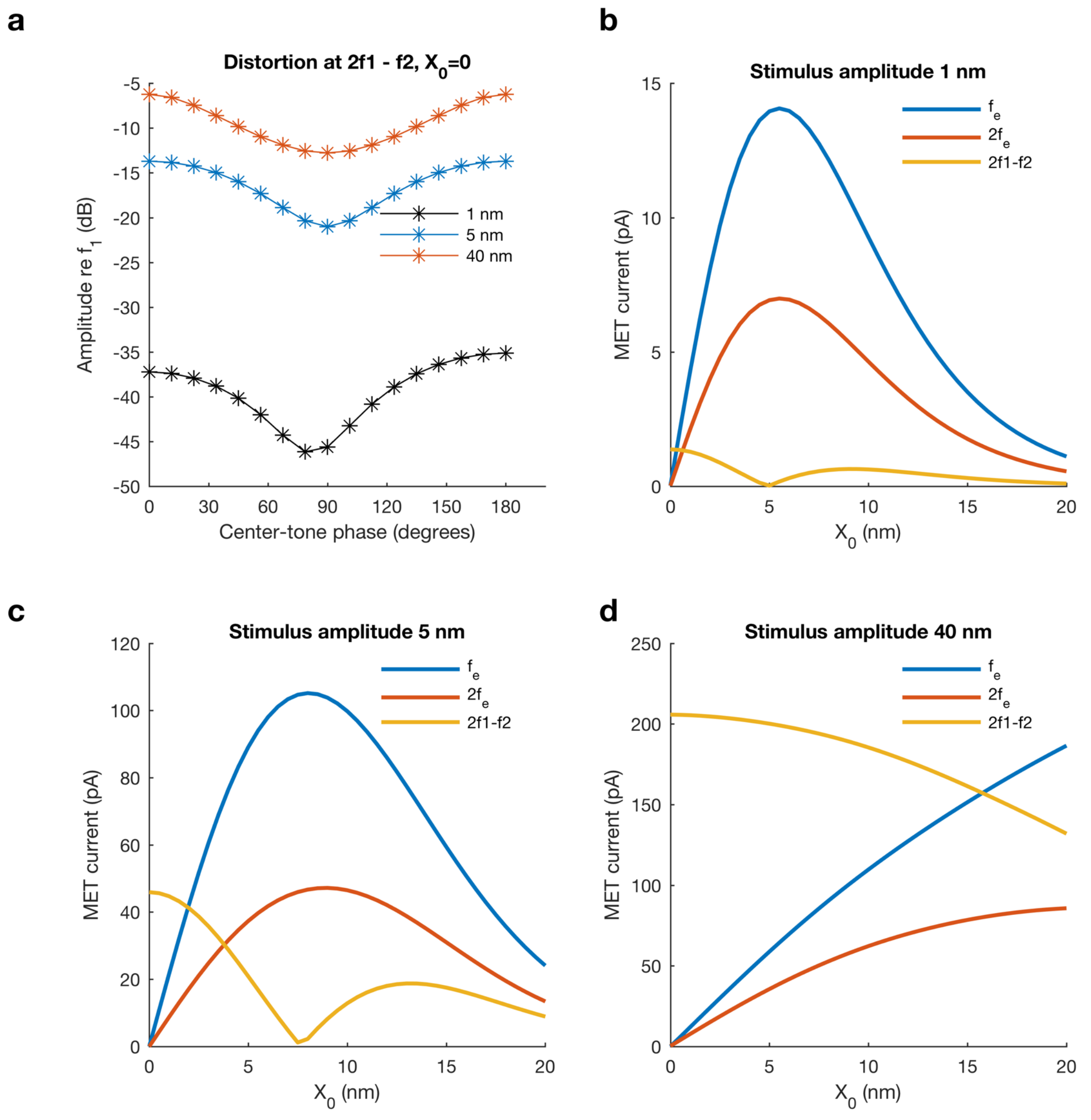

Suppl. Fig. 2. Distortion in transducer channel model. a. Relative level of the 2f1-f2 peak for different center-tone phases and stimulus levels. **b** and **c**. For 1-nm stimuli (b) and 5-nm stimuli (c), the f_e peak (blue line) was maximal near a minimum for the 2f1-f2 peak (yellow line). **d**. Qualitatively different behaviour for saturating stimuli. In all four graphs, model parameters are identical to those used in Fig. 7 of the main text. f_1, frequency of the first primary tone, see also Eq. 1 in the main text; MET, mechanoelectrical transducer.
